# Retrospective Study of Normal Variations in Vertebral Artery on Computed Tomography Angiography With Special Emphasis on Relevant Embryology

**DOI:** 10.7759/cureus.38063

**Published:** 2023-04-24

**Authors:** Pranjal Phukan, Bishwajeet Saikia, Amitav Sarma, Sudipta D Baruah, Gautam C Das, Partha S Gayan

**Affiliations:** 1 Radiology, North Eastern Indira Gandhi Regional Institute of Health and Medical Science (NEIGRIHMS), Shillong, IND; 2 Anatomy, North Eastern Indira Gandhi Regional Institute of Health and Medical Science (NEIGRIHMS), Shillong, IND; 3 Anatomy, Silchar Medical College and Hospital, Silchar, IND; 4 Ophthalmology, Nagaon Medical College and Hospital, Nagaon, IND

**Keywords:** vascular variations, arterial embryology, transient ischemic attack, cerebrovascular circulation, computerized tomography, brain anatomy, vertebral artery (va)

## Abstract

Background

The vertebral arteries (VA) nourish the posterior cerebral circulation. Planning neck and cervical interventions like drilling and instrumentation, which involves VA manipulation, require an in-depth acquaintance with the normal and variant patterns encountered in the origin and course of the VA. Embryological events involved in forming these variant patterns can be correlated to their prior disposition in the lower vertebrate's understanding which becomes crucial while planning cervical interventions.

Study design

This is a single-center, retrospective study.

Materials and methods

The study involved 70 patients of both sexes and was done from September 2021 to February 2022 in the Department of Radiodiagnosis and Imaging at North Eastern Indira Gandhi Regional Institute of Health and Medical Sciences (NEIGRIHMS), Meghalaya, India. The CT angiographies were studied for variations of VA under V1 - from origin to entrance into the foramen transversarium (FT), V2 - part inside FT, V3 - from its exit from FT till it pierces the cranial dura mater, and V4 - intracranial part. Further, VA was observed for its origin, dominance, level of entry in FT, and any associated anomalies.

Results

The VA was found mostly to be codominant. There was an opposite directional relationship between the basilar artery curvature and the dominance of VA. The association of ischemic events with hypoplastic VA was more on the left side (66.67%). Left VA originated from the aorta in 4.3% of subjects. One case presented a dual origin of VA. The abnormal origin of the LVA from the aorta showed a higher rate of abnormal entry into FT which was also found to be statistically significant.

Conclusion

Our study identifies and documents the anatomical variations present in VA specific to the population of northeast India by CT angiography and thus provides a much-needed reference for the healthcare professionals working in the field of Head and Neck interventions by providing opportunities further to understand these patterns for better diagnostic and therapeutic outcomes.

## Introduction

The anatomy of the blood vessels supplying the brain becomes crucial during episodes of vascular compromise. The posterior cerebral circulation is essentially nourished by the two vertebral arteries (VA), which originated as the first branch from the subclavian artery at the root of the neck. The origin of the VA and its level of entry into the foramen transversarium is fairly common [1]. During embryonic development, these arteries may be influenced by triggers that may alter the vascular construction program and can give rise to variations. Alternatively, some variations may represent the persistent embryological patterns in humans and may even represent primitive forms encountered in lower animals [2]. The variations in the VA are usually incidental findings without any clinical symptoms [3]. Critical knowledge of these variant patterns, often related to their development, becomes a prerequisite for a precise diagnosis and interventions in the cervical spine [3]. Our study attempts to identify and document the variations of VA and critically correlate the embryological events producing such variations in the population of Northeast India using CT angiography.

Development of VA

In the early stages of development, the neural tube gets its nutrition through the process of diffusion directly or through meninx primitiva [4]. With the further enlargement of the neural tube, intrinsic angiogenesis is triggered, which soon cascades new channels to establish communication with the developing perineural vascular network. During the fifth week of intrauterine life, the perineural arterial network establishes communication with the developing cardiac system [4]. The development of the aortic arches brings about the carotid arteries extending bilaterally till the ventral end of the prosencephalon [5]. By this time, a pair of arterial plexuses can be seen along the ventral wall of the hindbrain, the longitudinal neural artery (LNA). The LNA is the forerunner of the VA and the basilar artery (BA). Simultaneously the carotid arteries soon establish their communication with the system of LNA and irrigate them via some transient anastomotic arterial channels, namely, the trigeminal artery (TA), otic artery (OA), hypoglossal artery (HA), and pro-atlantal artery (pro-A). These channels represent primitive carotid-vertebral anastomosis [6,7,8].

The brain vesicles' growth produces significant changes in the cerebral vascular tree, where the carotid system divides into two branches near the prosencephalon's ventral aspect: a rostral telencephalic branch and a caudal communicating branch. The rostral branch will give rise to the future anterior cerebral and the anterior choroidal artery. In contrast, the caudal branch forms the posterior communicating artery (PCoA) and provides provision for the developing carotid system to communicate with the LNA [4]. Following this newly established communication, the pre-existing transient carotid-vertebral establishments regress [5]. The paired LNA later fuses in the midline to form the BA, thus completing the posterior circulation of the circle of Willis (COW) [4]. By the end of six weeks, the cerebral vascular tree nearly resembles its adult configuration. Simultaneously dorso-ventral communication can also be seen between the segmental arteries in the lower neck. These segmental arteries are, in fact, inter-segmental as they pass in between the embryonic somites. Soon these dorso-ventral communications regress and the first segmental artery supplies the system of LNA [4]. The disappearance of dorso-ventral anastomosis in the neck is followed by reinforcement of the longitudinal anastomosis between six and seven consecutive inter-segmental arteries giving rise to the VA. Thus, the VA distally supplies the vascular plexus of LNA, which is the future BA, and gains the vascular flow proximally by the seventh segmental artery, the future subclavian artery. The communication between the dorsal aorta and the LNA, i.e., the VA, now replaces the role of the distal carotid-vertebral connections as feeders to the posterior circulation. Although there is an HA regression, the pro-A artery's distal portion remains the transverse suboccipital part of the VA [6]. Agenesis at any site of these normally present segments or the persistence of their embryonic connections can result in specific variations observed in VA.

This article was previously posted to the ResearchSquare preprint server on 04/01/23.

## Materials and methods

The present retrospective study was done for a period of six months after due clearance from the Institution Ethics Committee, North Eastern Indira Gandhi Regional Institute of Health and Medical Sciences (NEIGRIHMS), Shillong, India (approval no. NEIGR/IEC/M15/F1/2021 dated 28/8/21). Out of 70 subjects observed, 18 subjects were female and 52 were male. The data was collected from September 2021 to February 2022 in the age groups ranging from 18 to 85 years. The indications for which the subjects underwent CT angiography were retrieved from the clinical notes and are listed in Table 1.

**Table 1:** Indications for CT angiography.

Indication of CT angiography	Number of patients
Intracranial hemorrhage	21
Trauma evaluation	19
Headache	14
Others (suspected vascular lesions)	16

Selection criteria

Inclusion Criteria

All adult subjects of both sexes belonging to different communities of Northeast India underwent head and neck CTA in the Department of Radiology, NEIGRIHMS.

Exclusion Criteria

All adult subjects with prior neck surgeries, inadequate scan coverage of head and neck region, missing demographic data, and patients who had undergone repeated scans.

Variations of VA

**Figure 1:**
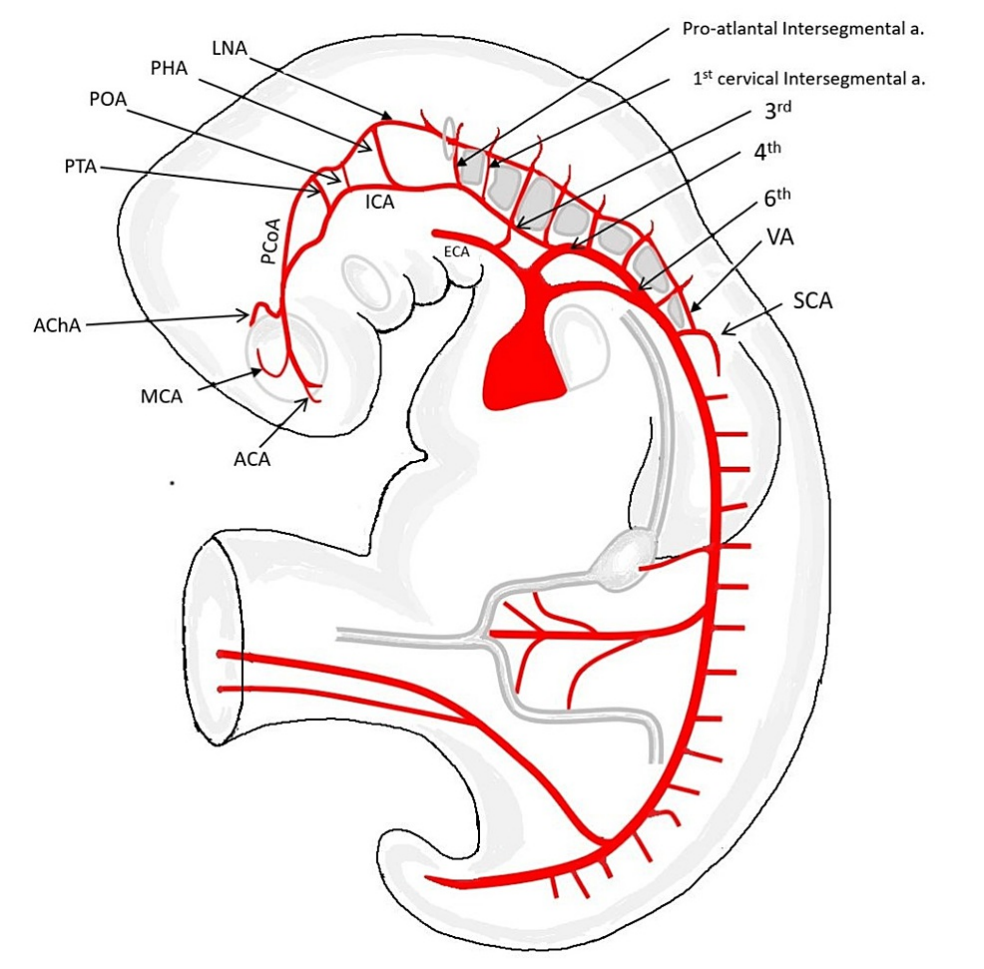
Schematic embryological representation showing the development of vertebral arteries and their relationship with the rest of the developing cerebral arterial system. ACA: Anterior cerebral artery; MCA: Middle cerebral artery; AChA: Anterior choroidal artery; PCoA: Posterior communicating artery; ICA: Internal carotid artery; ECA: External carotid artery; PTA: Primitive trigeminal artery; POA: Primitive otic artery; PHA: Primitive hypoglossal artery; LNA: Longitudinal neural artery; VA: Vertebral artery; SCA: Subclavian artery; 3rd: 3rd Pharyngeal arch artery; 4th: 4th Pharyngeal arch artery; 6th: 6th Pharyngeal arch artery. The figure was created by Dr. Bishwajeet Saikia.

**Figure 2:**
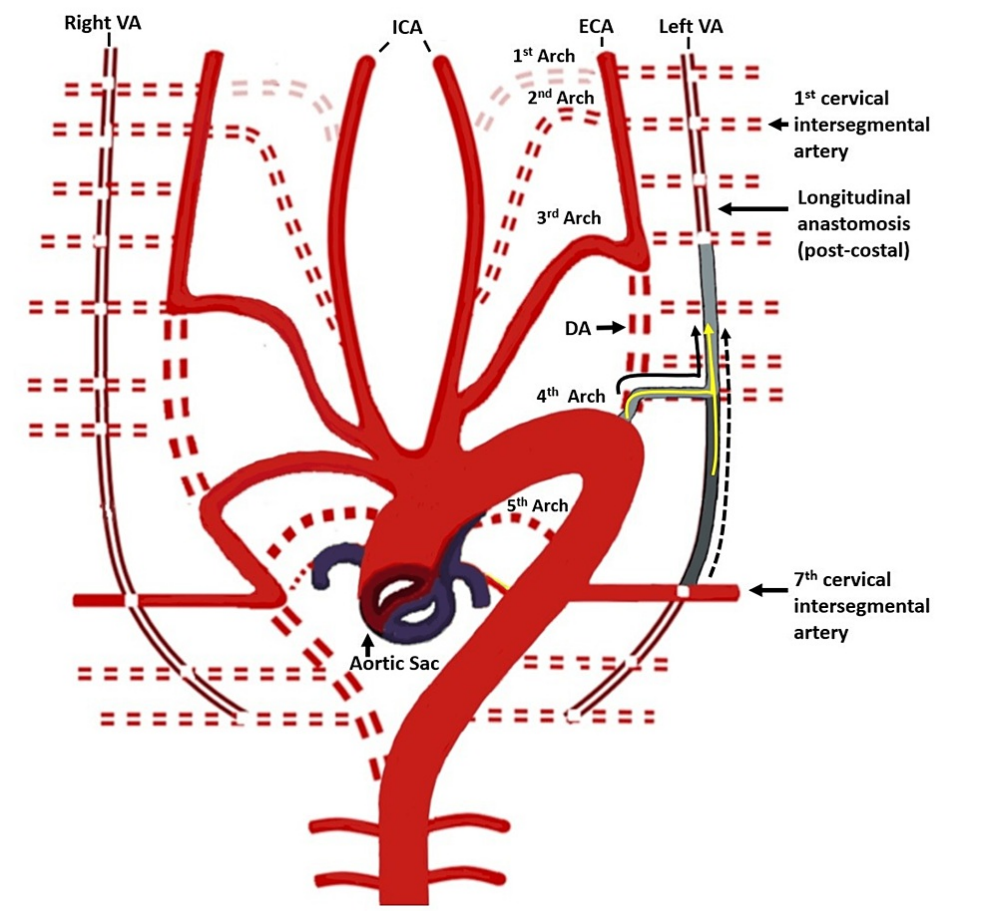
Schematic representation showing the normal (dotted black arrow), aortic origin (continuous black arrow from the 4th arch), and dual origin (yellow arrow) development of the vertebral arteries. VA: Vertebral artery; ICA: Internal carotid artery; ECA: External carotid artery; DA: Dorsal aorta. The figure was created by Dr. Bishwajeet Saikia.

Figure 1 below displays the embryological representation showing the development of VAs and their relationship with the rest of the developing cerebral arterial system. The development of the normal, aortic origin and dual origin of the VAs is shown in Figure 2. The VA was studied under V1: from origin to entrance into the foramen transversarium (FT); V2: part inside the FT; V3: from its exit from FT till it pierces the cranial Dura mater; V4: intracranial part. Further, the VA was observed for its origin, dominance, level of entry in the FT, and any associated anomalies like tortuosity and kinking. Normal origin was taken as the first branch of the subclavian artery (Figure 2). The dominance of VA was defined as a larger VA with a difference in diameter of ≥ 0.3 mm or VA connected with the BA in a straighter line [9-11]. The curvilinear angulation of the BA from its long axis can result from asymmetrical VA flow and is termed BA curvature. It is a morphological deformation related to areas of shear stress, stagnant flow, and clot formation. The dominance of the VA and its relationship with the BA curvature was compared. A hypoplastic VA was defined as having a diameter of <3 mm [9-11].

CT image acquisition and analysis

CTA images were obtained in 64 slice SIEMENS CT scan machine, and scanning parameters were 226 mA tube current, 0.4 sec tube rotation time, 120 kVp tube voltage, and 2 mm section thickness. Both pre-and post-contrast scans were obtained. A 60 ml of iodinated contrast medium was intravenously administered using a power injector at a rate of 4 ml/s and followed by a 35 ml saline chase. The region of interest was placed in ascending aorta, and image acquisition started at 12 sec. Post-contrast images were reconstructed for further processing. The images were transferred to picture archiving and communication systems (PACS) for image interpretation and comparison. One practicing neuroradiologist reviewed the images and did the reporting.

## Results

Our study found that most patients had a codominant pattern of VA (37, 52.85%). The dominance of VA on the left side was slightly more (18, 25.7%) than on the right (15, 21.42%) (Table 2). Further, it was found that most patients had an opposite directional relationship between the BA curvature and the dominance of VA (Table 2). The patients with codominant VA were seen mostly with a right-sided BA curvature. Hypoplastic VA was found on the right side in eight patients (11.4%) and three (4.3%) on the left side.

**Table 2:** The frequency of normal and variable anatomical patterns in origin, dominance, entry into FT, BA curvature, VA hypoplasia, tortuosity, and kinking. RD: Right dominance of VA; LD: Left dominance of VA; CoD: Codominance of VA; RC: Right curvature of the basilar artery; LC: Left curvature of the basilar artery; CC: Centralized basilar artery; RVA: Right vertebral artery; LVA: Left vertebral artery; IE: Ischemic events; SC: Subclavian artery; Ao: Aorta; B/L: Bilateral; TI: Type I tortuosity; II: Type II tortuosity; III: Type IV tortuosity; C4: 4th cervical vertebra; C5: 5th cervical vertebra; C6: 6th cervical vertebra.

Anatomical patterns/variables	Side	Observations	Percentage
Origin	RVA	SC	100%
		Ao	NIL
		Dual	NIL
	LVA	SC	95.7%
		Ao	4.3%
		Dual	1.4%
Dominance	RD	---	21.42%
	LD	---	25.7%
	CoD	---	52.85%
Entry into FT	RVA	C4	NIL
		C5	1.4%
		C6	98.6%
	LVA	C4	1.4%
		C5	1.4%
		C6	97.2%
BA Curvature	RD	RC	26.7%
		LC	66.7%
		CC	6.6%
	LD	RC	61.1%
		LC	7.8%
		CC	11.1%
	CoD	RC	59.5%
		LC	13.5%
		CC	27%
Hypoplastic VA	RVA	Total	11.4%
		With IE	25%
	LVA	Total	4.3%
		With IE	66.67%
Tortuosity	RVA	I	18.6%
		II	4.3%
		III	NIL
	LVA	I	20%
		II	5.7%
		III	NIL
	BVA	I	8.6%
		II	NIL
		III	NIL
Kinking	RVA	V1	4.3%
		V2	5.7%
		V3	NIL
		V4	1.4%
	LVA	V1	10%
		V2	1.4%
		V3	NIL
		V4	NIL

**Figure 3:**
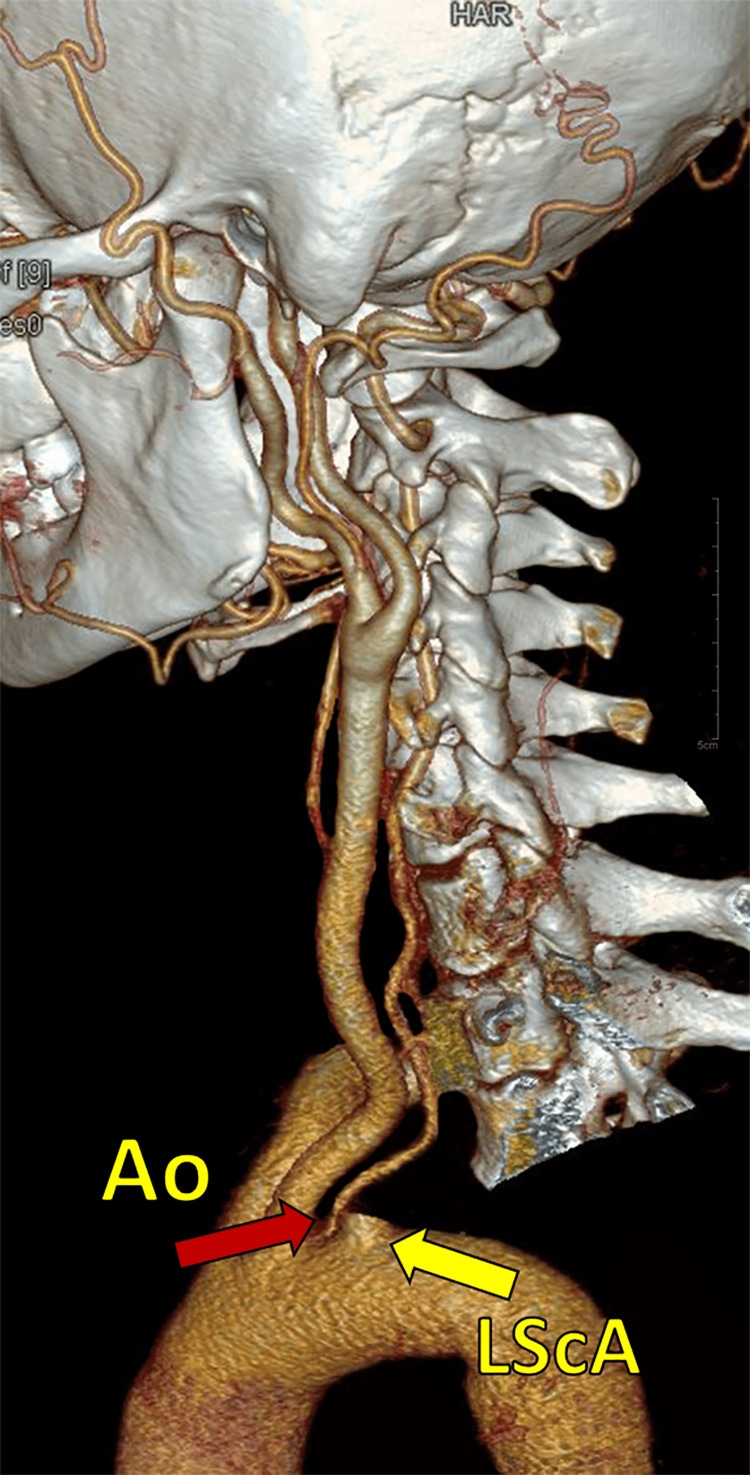
CT angiography 3D reconstruction image showing aortic origin (red arrow, Ao) of the left vertebral artery. The origin from the left subclavian artery has been cut in the image (yellow arrow, LScA) for better visualization of the aortic origin.

The association of ischemic events with VA hypoplasia was found more on the left side (66.67%) when compared to the right (25%) (Table 2). The right VA originated from the right subclavian artery in all subjects. On the left side, the VA originated from the left subclavian artery in 67 cases (95.7%). In three cases, the left VA originated from the aorta (4.3%) (Figure 3).

**Figure 4:**
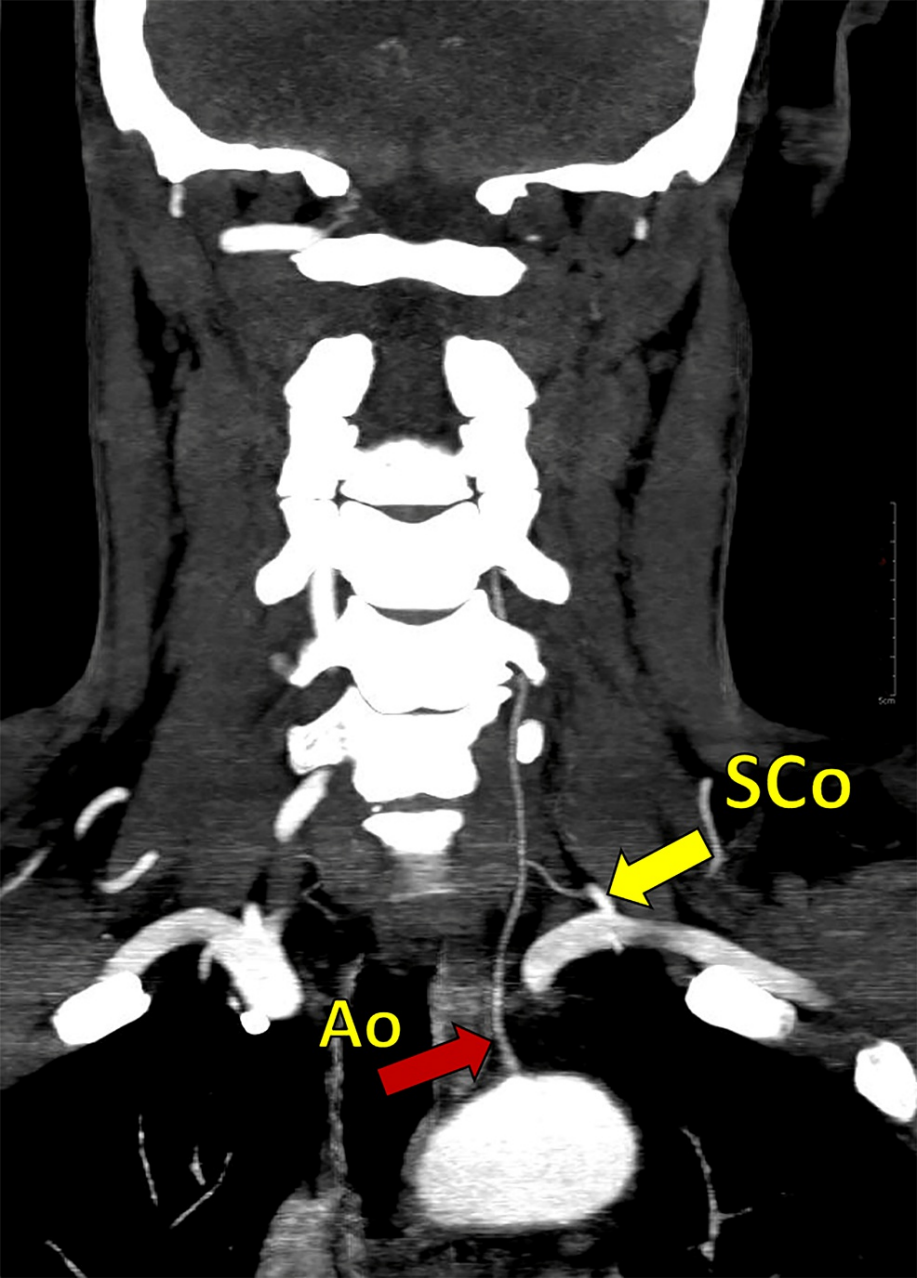
CT angiographic image showing dual origin (aortic: red arrow, Ao; subclavian origin: yellow arrow, SCo) of the vertebral artery on the left side. Note: Both origins were hypoplastic.

Out of these three cases mentioned, one (1.4%) presented with the dual origin of VA with an extra origin from the left-sided subclavian artery (Figure 4). On the right side, in one patient (1.4%), a VA normally originating from the subclavian artery was found to be entering from the FT of the 5th cervical vertebra. In contrast, the rest of the right-sided VA entered from its usual site (FT of the 6th cervical vertebra). On the left side, in one case, the VA, which originated as a normal branch of the subclavian artery, entered abnormally from the FT of the 5th cervical vertebra. For the dual origin of the VA on the left side, as mentioned earlier (Figure 4), the subclavian origin was seen to be entering the FT of the 6th cervical vertebra. In contrast, the aortic origin had an extra-vertebral course, and it entered the FT of the 4th cervical vertebra. Both these two origins were observed to be fused at the level of the 4th cervical vertebra (Figure 4). The abnormal origin of the LVA from the aorta, which was found in three cases, presented a higher rate of anomalous entry into the FT, and this phenomenon was found to be statistically significant (p < 0.005) (Fisher's exact test). Table 2 shows the kinking of the VA, which was found maximum on the left side (7, 10%) at the V1 level. There were no cases of fenestration observed in the study.

## Discussion

The VA diameters are often found to be unequal. It is also seen that an asymmetry in the VA flow pattern can influence the morphological deformation, like angular deviation of the BA from its long axis or BA curvature. These deformations may further influence the wall shear stress and the blood flow velocity inducing infarct formation. In our study, the BA curvature was found to be mostly opposite to the direction of the dominant VA, consistent with some previous findings [12]. Further, we found a dominance of right VA in 21.42% of patients, among which the BA curved to the left side in 66.7%; on the left, a dominance of VA was seen in 25.7% of patients, among which BA curved to the right side in 61.1%. The asymmetric flow pattern arising from such architecture can give rise to peri-vertebrobasilar (pontine, posterior inferior cerebral artery) junctional infarcts [12]. The left-sided VA dominance is a common finding. Hypoplasia can be congenital or acquired, and they are required to be differentiated for investigating the flow hemodynamics. A segmental narrowing in VA may represent an inability to reinforce the longitudinal anastomosis between consecutive inter-segmental arteries giving rise to the VA (Figure 2) [2]. Hypoplastic VA is seldom considered an independent risk factor for a stroke. However, it was recently suggested that additional risk factors of vascular compromise may precipitate ischemic events [13]. In our study, the association of ischemic events in the posterior circulation and VA hypoplasia was found in 66.7% of the subjects on the left and in 25% of subjects on the right side (Table 2). Even in the pediatric age group, VA hypoplasia may be a prominent cause of transient ischemic attacks (TIA). These types of hypoplastic vessels can increase the child's susceptibility to arteriosclerosis later in life. However, in our study, none of the subjects was of pediatric age group to correlate the same [14, 15]. Chen X et al. reported a hypoplastic right VA in 5.9% of patients and a hypoplastic left VA in 3.3% [16]. In our study, we observed a hypoplastic right VA in 11.4% of patients and a hypoplastic left VA in 4.3%. Anomalies in the origin of the VA can be related to their embryological development. The origin of VA from the aorta was reported earlier [17, 18]. This developmental anomaly arises when the 6th dorsal intersegmental artery with its dorsal branch and the adjacent segment of the dorsal aorta fails to disappear. With a persistent blood flow in this segment, the VA soon gains its origin from the aorta. The newly redirected flow simultaneously regresses the classical segment for normal VA origin, which eventually disappears due to the subsequent flow reduction. In our study, we found three cases of aortic origin from the left VA (Figure 3), out of which one case presented with an additional origin of VA, which was normal and was seen to be arising as the first branch of the subclavian artery. This whole arrangement presented as a dual origin of VA on the left side. Duplication in the origin of the left VA is rare and refers to VA with two origins that fuse at various levels [17, 19-22]. The persistence of the embryological intersegmental arteries, along with the normal subclavian origin, can lead to such configurations (Figure 2). This aortic origin may produce greater shear stress compared to normally arising subclavian VA, as it receives a direct pulsatile blood flow compared to the dampening effect created in the latter [23]. Consequently, the aortic origin in VA is reported to be associated with a higher incidence of VA dissection, aneurysms, kinking, and arterio-venous malformations [21, 24]. Contrary to this, we found kinking of the subclavian origin instead of aortic in the left dual origin of VA. An abnormal aortic origin of the right VA is usually rare, ranging from 0.6% to 0.19% [25-27]. Dual origin on the right side is even rare and was reported in as low as 0.1% of patients [21]. The present study found no abnormality in the origin of VA on the right side (Table 3). Left VA originating from the aorta is comparatively frequent, ranging from 2.7% to 6.3% [21, 25, 26]. The aortic origin of VA on the left side was observed in three cases, out of which one such case (1.4%) had a dual origin. Dual origin of left VA is a rare finding and was reported earlier in 0.3% of patients (Table 3) [21].

**Table 3:** Comparison of VA dominance, BA curvature, VA hypoplasia, origin, and entry into the FT. RD: Right dominance of VA; LD: Left dominance of VA; CoD: Codominance of VA; RC: Right curvature of the basilar artery; LC: Left curvature of the basilar artery; CC: Centralized basilar artery; RVA: Right vertebral artery; LVA: Left vertebral artery; IE: Ischemic events; SC: Subclavian artery; Ao: Aorta; FT: foramen transversarium.

Anatomical patterns			Our study	Hong JM et al. [12]		Chen X et al. [16]	Meila D et al. [25]	Lin C et al. [26]	Komiyama M et al. [21]	
Dominance	RD		21.42%	31.6%		----	----	----	----	
	LD		25.7%	68.4%		----	----	----	----	
	CoD		52.85%	NIL		----	----	----	----	
BA curvature	RD	RC	26.7%	---	--	----	----	----	----	
		LC	66.7%			----	----	----	----	
		CC	6.6%			----	----	----	----	
	LD	RC	61.1%	---	--	----	----	----	----	
		LC	27.8%			----	----	----	----	
		CC	11.1%			----	----	----	----	
	CoD	RC	59.5%	---	--	----	----	----	----	
		LC	13.5%			----	----	----	----	
		CC	27%			----	----	----	----	
Hypoplastic VA	RVA	%	11.4%	----		5.9%	----	----	----	
										LVA	%	4.3%	----		3.3%	----	----	----	
	Origin	RVA	SC	11.4%	----		----	99.8%	99.2%	100%	
Ao											NIL	----		----	0.19%	0.6%	NIL	
Dual			NIL	----		----			0.1%	
LVA			SC	95.7%	----		----	94.2%	96.3%	97.3%	
		Ao	4.3%	----		----	6.3%	3.7%	2.7%	
		Dual	1.4%	----		----	----	----	0.3%	
Entry into FT		RVA	C4	NIL	----		----	1.3%	1.5%	2.2%	
	C5										1.4%	----		----	6.3%	2.5%	3.3%	
	C6		98.6%	----		----	84.8%	96%	94.3%	
	LVA		C4	1.4%	----		----	1.3%	0.6%	1.5%	
		C5	1.4%	----		----	4.5%	4.2%	6.5%	
		C6	97.2%	----		----	86.1%	95.2%	91.5%	

**Figure 5:**
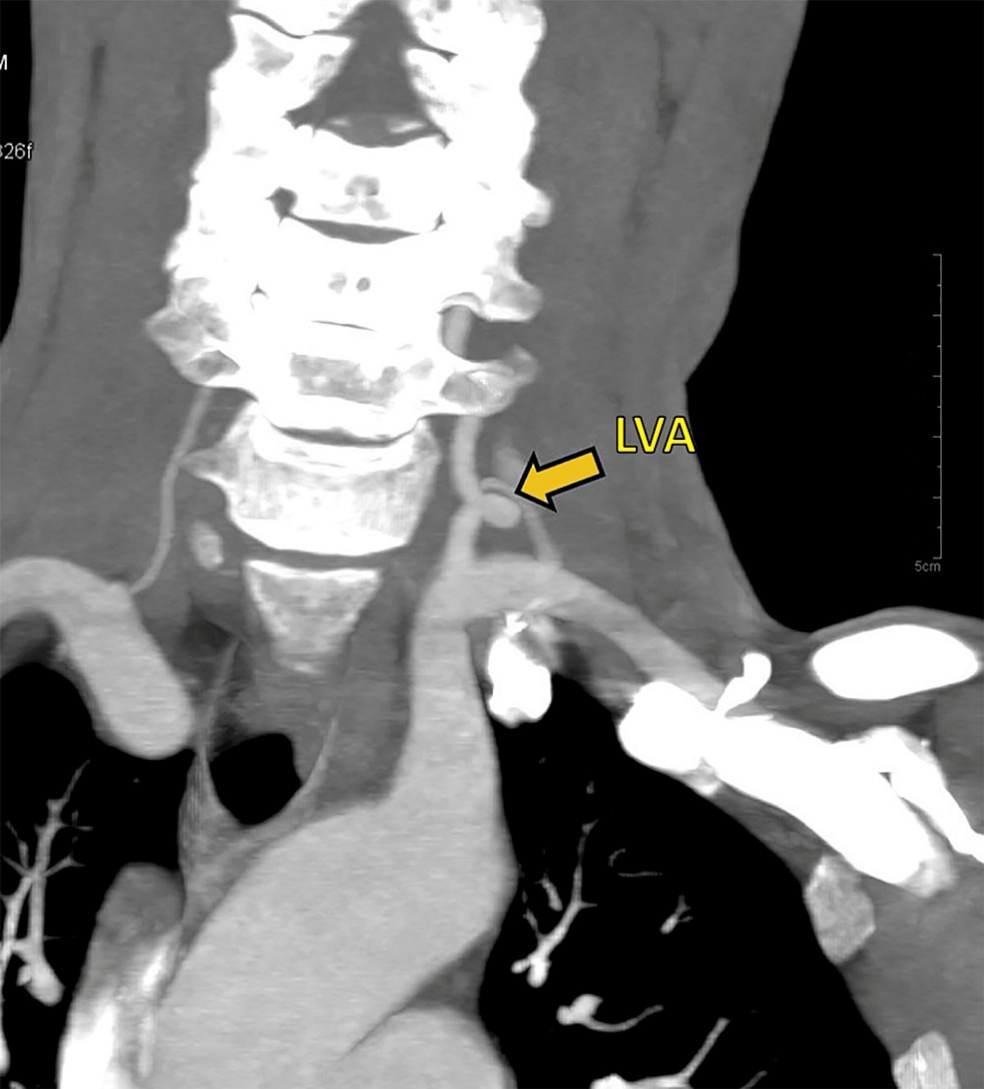
CT angiographic image showing grade III tortuosity of left vertebral artery (yellow arrow, LVA) with a hypoplastic right-sided VA.

Embryological events which can lead to the persistence of a particular intersegmental artery decide the level at which the VA originating from the aorta will enter the respective FT in adult life [22]. Abnormal entry into the FT was found to be as high as 2.2% on the right-sided 4th cervical vertebra and 6.5% on the left-sided 5th cervical vertebra. The present study found an abnormal entry of VA in one case (1.4%) at the 5th cervical vertebral FT level on the right side and one (1.4%) on the left at the level of the 4th cervical vertebral FT (Table 3) [21, 25, 26]. Consistent with some previous studies, we further observed that an anomalous aortic origin of VA was invariably associated with an abnormal entry into the FT [25]. We found that the abnormal origin of the LVA from the aorta was presented with a higher rate of anomalous entry. This phenomenon was found to be statistically significant (p < 0.005) (Fisher's exact test). Kinking of VA is rare, while tortuosity is uncommon [27]. The exact etiology and pathophysiology of kinking and tortuosity are unknown; however, long-term hypertension, atherosclerosis, and aging can be attributed to it [28]. In children and young adults, tortuosity and kinking are mostly considered to be congenital. Hemodynamic instabilities arising from arterial tortuosity may cause vertigo, and in kinking, it may lead to symptoms of TIA [28]. In our study, the tortuosity of VA was observed mostly on the right side, with maximum tortuosity observed in the V1 segment. Kinking was observed more in the left-sided VA (Figure 5) in seven cases (10%), while the most affected segment was V2 on the right side. Clinical presentation of headache was associated with two cases where kinking and tortuosity were observed.

Limitations

The present study highlights the most common variant patterns and the relevant embryological events producing them in the population of Northeast India. The retrospective nature of this study may have led to a selection bias. More multicentric studies with larger sample sizes are required for further evidence.

## Conclusions

Anomalous patterns observed in the VA may affect the posterior circulation and are often incidental findings during a routine pre- or intra-operative cervical procedure. The present study highlights the major variant patterns in the extracranial course of the VA and critically correlates them with their embryological development. The results are specific to the population of Northeast India and thus provide a much-needed regional reference. Further, understanding the embryological events and the hemodynamic changes in these variant patterns becomes crucial for better outcomes while planning diagnostic or therapeutic interventions of the head and neck.
